# A shortest-path graph kernel for estimating gene product semantic similarity

**DOI:** 10.1186/2041-1480-2-3

**Published:** 2011-07-29

**Authors:** Marco A Alvarez, Xiaojun Qi, Changhui Yan

**Affiliations:** 1 Department of Computer Science, Utah State University, Logan, 84322, USA; 2 Department of Computer Science, North Dakota State University, Fargo, 58108, USA

## Abstract

**Background:**

Existing methods for calculating semantic similarity between gene products using the Gene Ontology (GO) often rely on external resources, which are not part of the ontology. Consequently, changes in these external resources like biased term distribution caused by shifting of hot research topics, will affect the calculation of semantic similarity. One way to avoid this problem is to use semantic methods that are "intrinsic" to the ontology, i.e. independent of external knowledge.

**Results:**

We present a shortest-path graph kernel (spgk) method that relies exclusively on the GO and its structure. In spgk, a gene product is represented by an induced subgraph of the GO, which consists of all the GO terms annotating it. Then a shortest-path graph kernel is used to compute the similarity between two graphs. In a comprehensive evaluation using a benchmark dataset, spgk compares favorably with other methods that depend on external resources. Compared with simUI, a method that is also intrinsic to GO, spgk achieves slightly better results on the benchmark dataset. Statistical tests show that the improvement is significant when the resolution and EC similarity correlation coefficient are used to measure the performance, but is insignificant when the Pfam similarity correlation coefficient is used.

**Conclusions:**

Spgk uses a graph kernel method in polynomial time to exploit the structure of the GO to calculate semantic similarity between gene products. It provides an alternative to both methods that use external resources and "intrinsic" methods with comparable performance.

## Background

The Gene Ontology (GO) [1] systematically organizes knowledge by means of well-structured controlled vocabularies and provides consistent descriptions to organisms across species. GO terms have been widely used to annotate genes and gene products in the Gene Ontology Annotation (GOA) project [2]. As the GO becomes more and more important in biomedical research, computational methods are often needed to explore the GO to calculate the semantic similarity between gene products. Such methods have been used in a broad range of applications, including: clustering of genes in pathways [3-6], prediction of protein-protein interactions [7], and the evaluation of similarity between gene products with respect to expression profiles [8], protein sequence [9-11], protein function [12], and protein family [13].

The semantic similarity between two gene products is usually calculated based on the term similarity. First, pairwise semantic similarities between GO terms that annotate the gene products are calculated. Then, the these pairwise similarities are combined to derive an overall semantic similarity between the gene products. Different methods have been used to combine pairwise GO term similarities in previous research [4,8,10,11,14,15]. A representative collection of methods for calculating the semantic similarity between GO terms has been reviewed in [16]. Most of those methods use the information content (IC) of the nearest common ancestor (NCA) or most informative common ancestor (MICA) to quantify the amount of shared information between two GO terms. However, the IC is calculated based on the frequency of GO terms in external resources, such as GOA databases. External resources change as knowledge is updated (e.g., more annotations are included in GOA). Consequently, for the same pair of GO terms, their semantic similarity computed by these methods might change as the external resources evolve. However, semantic similarities between GO terms should not be affected by such changes. In addition, certain annotations might be frequent simply because of popular research topics, leading to biased results. Some other methods rely on distance measures [17,18], e.g. counting the number of edges on the shortest path between the involved terms in the GO, to compute the GO term similarity. One shortcoming of this approach is that the edges in the GO do not imply equal length in semantics. Although some methods tried to address this issue by assigning different weights to edges at different levels, they still suffer from the fact that GO terms at the same level do not necessarily have the same specificity. Other methods calculate the semantic similarity between gene products without considering the semantic similarity between GO terms. In these methods, a gene product is represented by a set or a vector of GO terms that annotate it. Then, the semantic similarity between gene products is calculated as the overlap between sets or the inner product of vectors [4,10]. However, these methods did not exploit the structure of the GO and ignored the relationship between GO terms.

To address the aforementioned issues, we propose a shortest-path graph kernel (spgk) method for calculating the semantic similarity between gene products. In spgk, each gene product is represented as a graph, which is an induced subgraph of the GO. Then a graph kernel method is used to calculate the semantic similarity between the graphs. Spgk is intrinsic to the GO, i.e., it does not rely on external resources to calculate the semantic similarity. Thus, it does not have the same drawbacks as the methods based on the IC of GO terms. At the same time, it uses a graph to explicitly explore the GO structure and exploit the relationship between GO terms. Graph matching is computationally expensive in general, being an NP-complete problem on general graphs. To reduce the computational complexity, we develop a graph kernel to calculate the similarity between graphs. Using a comprehensive evaluation benchmark developed by another group, we compare spgk with other state-of-the-art methods.

## Methods

In this section, we present a novel method for calculating the semantic similarity between proteins. First, we introduce basic background of the Gene Ontology. Then we describe the details of the graph kernel method.

### Gene ontology and gene ontology annotations

The GO project [1] maintains a dynamic, structured, precisely defined, and controlled vocabulary of terms for describing the properties of gene products across species. The GO consists of three different ontologies describing: 1) biological processes (BP), where a process often involves a chemical or physical transformation (e.g. cell growth); 2) molecular functions (MF), where functions are defined as the biochemical activity of gene products (e.g. enzymes); and 3) cellular components (CC), which refers to places in the cell where gene products are active (e.g. nuclear membrane). Each ontology is structured as a directed acyclic graph, where nodes (GO terms) are linked to each other through "is-a", "part-of" or "regulates" relationships. On the other hand, the annotation of gene products is the process of assigning ontology terms to gene products in order to describe their activities and localization. For example, the GOA project [2], at the European Bioinformatics Institute (EBI), aims to provide high-quality electronic and manual annotations to UniProt KnowledgeBase (UniProtKB) entries [19]. GOA annotations are obtained from strictly controlled methods, where every association is supported by a distinct evidence source. A protein can be annotated with multiple GO terms from any of the three ontologies in the GO. Functional annotations of UniProtKB proteins currently consist of over 32 million annotations, which cover more than 4 million proteins [2].

### Graph representation of proteins

**Figure 1:**
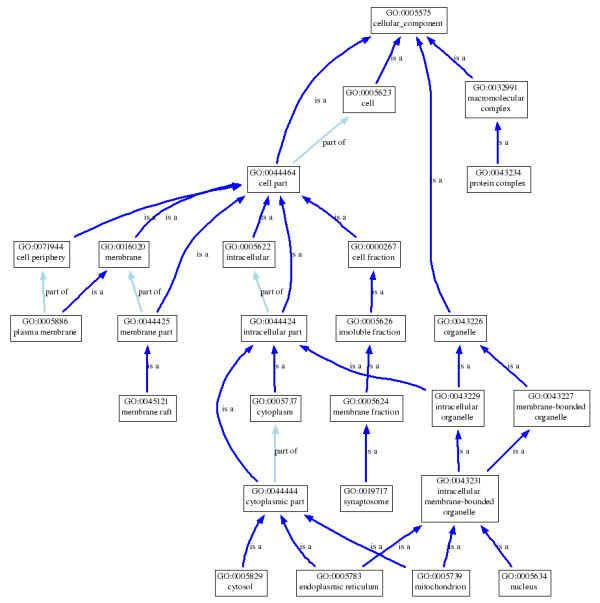
**A subgraph generated from GO**. The subgraph consisting of terms annotating protein P17252 (Protein kinase C alpha type) and their ancestors in the Cellular Component ontology.

We represented a protein using a subgraph of the ontology that consisted of all the GO terms annotating the protein and their ancestors in the ontology. Each edge of the graph corresponds to a relationship between two terms in the ontology. There are three types of relations in the GO: is-a, part-of, and regulates. Since the GO includes three different ontologies, the resulting graph will be different when a different ontology is used. For example, Figure 1 shows the graph generated for UniprotKB protein P17252, using the Cellular Component (CC) ontology.

### A shortest-path graph kernel for proteins

We used a shortest-path graph kernel to compare two graphs as proposed in [20]. First, let's define the shortest-path graph. Given a graph *G = (V, E)*, its shortest-path graph is *G_sp _*= *(V, E')*, where *E' = {e'_1_,...,e'_l_} *such that *e'_i _*= *(u, v)*, where *u *∈ *V*, *v *∈ *V*, and *path(u, v)*≠0. That is, *G_sp _*has the same vertices as G and the edge *(u, v) *in *G_sp _*has the same length as the shortest distance between *u *and *v *in *G*. This transformation can be performed using any all-pairs shortest path algorithm. In particular, the Floyd-Warshall algorithm is used in spgk because it is straightforward and has time complexity of O(n^3^). Then, for a pair of graphs, the shortest-path kernel calculates their similarity by comparing every pair of edges in their shortest-path graphs. For example, Let *G_1 _*= *(V_1_, E_1_) *and *G_2 _*= *(V_2_, E_2_) *be two graphs and *G_1sp _= (V_1_, E'_1_) *and *G_2sp _= (V_2_*, *E'_2_) *be their shortest-path graphs respectively. The similarity between *G_1 _*and *G_2 _*can be calculated using Eq. 1.(1)

where *k_walk _*is a positive definite kernel for comparing two walks. In this case, a walk includes an edge and its two end nodes. Let *e_1 _*be the edge connecting nodes *v_1 _*and *w_1_*, and *e_2 _*be the edge connecting nodes *v_2 _*and *w_2_*, then *k_walk_(e_1_, e_2_) *is defined by Eq. 2.(2)

where *k_node _*is a kernel function for comparing two nodes, which returns 1 when the two nodes are identical and 0 otherwise, and *k_edge _*is a kernel function for comparing two edges. *k_edge _*is a Brownian bridge kernel that returns the largest value when two edges have identical length, and 0 when the edges differ in length more than a constant *c *as shown in Eq. 3. In this study, we use *c = 2 *as suggested by [20].(3)

### Evaluation approach

We evaluated the performance of spgk by comparing the resulting semantic similarities with protein functional similarities derived from expert annotations. Functional similarities between proteins were derived from the Pfam database [21] as described by Couto et al. [13]. Let P denote a protein and *F(P) = *{*f_1_, f_2_,..., f_n_*} be the set of Pfam families that P is associated with. Then the functional similarity between two proteins *P_i _*and *P_j _*is given by Eq. 4(4)

Previous study by Xu et al. [7] shows that having more annotations per protein in the dataset leads to more reliable functional similarity estimation from the GO. Thus, for the purpose of evaluation, we carefully selected a set of 100 proteins from GOA, such that they were the top 100 proteins with the highest numbers of annotations. We also ensured that for any selected protein: 1) it existed in the UniProtKB/Swiss-Prot database, 2) it had at least one annotation from each of the three ontologies in GOA-Uniprot, and 3) it had at least one Pfam-A annotation. The evaluation proceeded as follows: First, the graph kernel was used to calculate pairwise semantic similarities for a set of proteins. Second, pairwise functional similarities between the proteins were calculated based on the Pfam database annotations. Last, the Pearson's Correlation Coefficient between the semantic and functional similarities was calculated. If two proteins have similar function, then a good semantic similarity method should detect high semantic similarity between them. Thus, higher values of Pearson's Correlation Coefficient indicate better performance in the calculation of the semantic similarity. This procedure was repeated for each of the three ontologies in the GO, namely, BP, MF, and CC.

## Results and discussion

### Datasets

In our experiments, we used the revision 1.723 of the GO and the release 74.0 of GOA-Uniprot, where GO terms are assigned to proteins in UniProtKB by manual and electronic methods [2]. As mentioned before, the GO contains three different ontologies that describe gene products in terms of their associated biological processes, molecular functions, and cellular components.

### Performance of spgk

100 proteins with the most GOA annotations were selected as described in the Methods section. Spgk was used to calculate pairwise semantic similarities between the proteins. The correlation coefficient between the resulting semantic and functional similarities was calculated. The evaluation was repeated using three different ontologies of the GO. The results are shown in Table 1 which reveals a couple of interesting points. First, spgk produces semantic similarities that are highly correlated with functional similarities for all three ontologies. Second, when the CC ontology is used, the correlation coefficients are lower than when the MF and BP ontologies are used. This is not surprising because the MF and BP ontologies are directly related to functions while the CC ontology is related to cellular components and locations.

**Table 1:** Performance of spgk.

Ontology	BP	MF	CC
Pearson's Correlation Coefficient	0.855	0.852	0.703

The performance is measured by the Pearson's correlation coefficients between the semantic similarity given by spgk and the functional similarity estimated from Pfam annotations. BP, MF, CC are the three ontologies in the GO.

### Comparison of spgk with state-of-the-art methods

To compare spgk with other existing methods, we used the Collaborative Evaluation of GO-Based Semantic Similarity Measures (CESSM) online tool [22]. This tool has been made available by the XLDB research group at the University of Lisbon. For the purpose of comparisons, CESSM provides a standard dataset consisting of 13,340 pairs of proteins involving 1,039 distinct proteins and implements 11 state-of-the-art semantic similarity methods, namely, simGIC and simUI [9], and three versions (the average, maximum and best-match average) of three different term similarity methods, namely Resnik [23], Lin [24], and Jiang & Conrath [25]. As a result, users can compare their methods with the 11 methods using the standard dataset.

As pointed out by Pesquita et al. [9] in a comprehensive evaluation, the maximum and average versions of term similarity methods have limitations from a biological point of view. Comparisons using the standard datasets at CESSM also confirmed that the best-match average version has better performance than the maximum and average versions for Resnik [23], Lin [24] and Jiang & Conrath [25] methods. Thus, in this section, we will compare spgk with simGIC, simUI, and the best-match average version of Resnik [23], Lin [24] and Jiang & Conrath [25] methods using CESSM. CESSM provides three different ways for evaluating a semantic similarity method, i.e., comparing the resulting semantic similarities with (1) functional similarities measured as sequence similarities, (2) functional similarities derived from enzyme commission (EC) classification, and (3) functional similarities derived from Pfam annotations.

Since the MF ontology is more closely related to function than the BP and CC ontologies, we will use the MF ontology to compare different methods. As pointed out by Pesquita et al. [9], the relationship between the semantic similarity and the sequence similarity is not linear. Thus, they recommended to use resolution instead of correlation coefficient to evaluate how well the semantic similarity matches the sequence similarity. Based on their definition, resolution is the relative intensity where variations in the sequence similarity scale are translated into the semantic similarity scale. Higher resolution values mean that the semantic similarity method has a higher capability to distinguish between different levels of protein functions. Therefore, a method with a higher resolution performs better than a method with a lower resolution. Table 2 shows the resolutions for different methods when the sequence similarity is compared with the semantic similarity computed by the methods. When the semantic similarity is compared with the function similarity derived from the EC classification and Pfam annotations, the Pearson's correlation coefficient is used as described in Methods. Tables 3 and 4 show the results.

**Table 2:** Comparison I.

Method	Resolution
spgk	0.976
simUI	0.967
Resnik	0.958
simGIC	0.956
Lin	0.571
Jiang & Conrath	0.241

The performance is measured by the resolution score.

**Table 3:** Comparison II.

Method	EC Similarity
spgk	0.646
Lin	0.642
simUI	0.637
simGIC	0.622
Resnik	0.603
Jiang & Conrath	0.561

The performance is measured by the Pearson's correlation coefficient between the resulting semantic similarity and the functional similarity derived from the EC classification.

**Table 4:** Comparison III.

Method	Pfam Similarity
simGIC	0.638
spgk	0.622
simUI	0.618
Resnik	0.572
Lin	0.564
Jiang & Conrath	0.491

The performance is measured by the Pearson's correlation coefficient between the resulting semantic similarity and the functional similarity derived from Pfam annotations.

The spgk method achieves the best results in tables 2 and 3, and is the second best in table 4. In addition to the better performance, the key advantage of spgk is that it is intrinsic to the ontology, i.e., it does not rely on external resources in the calculation of the semantic similarity. In contrast, all the other methods (except simUI) shown in tables 2, 3 and 4, rely on external resources, i.e., the annotations in GOA. Despite the high computational cost associated with the general graph comparisons, spgk does not suffer from this drawback. Using the shortest-path graph kernel, spgk requires a polynomial time (*O(n^4^)*), where n is the number of vertices. In additioin, each step of the graph kernel is simple to compute. For example, *k_node _*only needs to compare whether two vertex IDs are identical, and *k_edge _*considers the length difference between two edges. Thus, the constant factors associated with the polynomial time complexity are very small and spgk can run very fast in real applications.

SimUI is also intrinsic to the ontology. In simUI, the semantic similarity between two proteins is defined as the fraction between the number of GO terms shared by the two proteins and the number of GO terms in their union. Thus, simUI requires only a linear time (*O(n)*) and has the advantage that it is simple and faster for calculation. However, tables 2, 3, 4 show that spgk slightly outperformed simUI in all cases. We estimated the statistical significance of the improvement of spgk over simUI using Fisher's transformation. The *p *values were less than 0.001 when resolution was used to measure performance (table 2), 0.0384 for the EC similarity correlation coefficient (table 3) and 0.2266 for the Pfam similarity correlation coefficient (table 4). Therefore, compared with the conventional threshold of 0.05, the improvement is significant when the performance is measured by resolution and EC similarity correlation coefficient, but is insignificant when measured by Pfam similarity correlation coefficient. Comparing tables 2, 3, 4, we can see that the performance in table 4 is the poorest for all the methods. That might partially explain why the improvement is insignificant when Pfam similarity correlation coefficient is used as the measurement (table 4).

## Conclusions

In this manuscript, we have presented a method (spgk) that computes the semantic similarity between gene products using only information intrinsic to GO. In comprehensive evaluations using a benchmark dataset, spgk compares favorably with other state-of-the-art methods that depend on external resources. Compared to simUI, spgk achieves slightly better results but also has a higher time complexity. A big difference between spgk and simUI is that spgk takes into account the structure of the ontology. Since the structure of the ontology contains important information, it is important to exploit them to capture semantic similarity. The results presented here show that spgk provides an alternative to both methods that rely on external resources and "intrinsic" methods with comparable performance.

In light of future development, there are still some limitations in spgk at its current form. For example, in spgk, the function (*k_node_*) that compares nodes only considers whether the two nodes are identical. However, each node in the GO is associated with a text definition, which contains rich information that is useful for deriving biological relationship between nodes. Thus, one direction for future improvement is to take into account the semantics of the text definition when comparing nodes. Furthermore, the *k_edge _*function only considers the length difference between two paths. In GO, the edges are associated with different types of relationship. Since different types of relationship have different biological meanings, they should be given different weights. Thus, another direction for improvement is to systematically explore weighting methods that assign different weights to the edges based on the biological relationships.

## Competing interests

The authors declare that they have no competing interests.

## Authors' contributions

CY conceived the project and supervised all aspects of the research. MA contributed to programming, discussion, data analysis and preparation of the first draft. XQ contributed to discussion. All authors read and approved the final manuscript.
